# A new species of *Pristimantis* (Amphibia, Anura, Craugastoridae) from a montane forest of the Pui Pui Protected Forest in central Peru (Región Junín)

**DOI:** 10.3897/zookeys.645.11221

**Published:** 2017-01-12

**Authors:** Edgar Lehr, Jiří Moravec

**Affiliations:** 1 Department of Biology, Illinois Wesleyan University, P.O. Box 2900, Bloomington, IL 61701, USA; 2 Department of Zoology, National Museum, Cirkusová 1740, 193 00 Prague 9, Czech Republic

**Keywords:** Andes, montane forests, anuran diversity, Pristimantis ashaninka new species, Peru

## Abstract

A new species of frog of the genus *Pristimantis* is described from a montane forest between 1700 and 1800 m a.s.l. of the Pui Pui Protected Forest (Región Junín) in central Peru. *Pristimantis ashaninka* **sp. n.** is described based on five adult females (snout–vent length 23.1–26.7 mm) and ten juveniles (snout-vent length 10.6–13.4). It differs from its congeners by having the skin on dorsum shagreen with many conical tubercles giving it a spinose appearance, lacking a tympanum, having groin, anterior and posterior surfaces of thighs uniformly grayish brown, and a pale bronze iris with fine black reticulations, a median reddish hint horizontally across iris, and a black narrow vertical streak from pupil across lower and upper half of iris. Among the Peruvian *Pristimantis* that lack a tympanum, *Pristimantis ashaninka* **sp. n.** is morphologically most similar to *Pristimantis lirellus*, *Pristimantis martiae*, and *Pristimantis rhabdocnemus*. However, 16S DNA barcoding revealed clear distinctions between all four species of *Pristimantis*.

## Introduction

**Figure 1.**
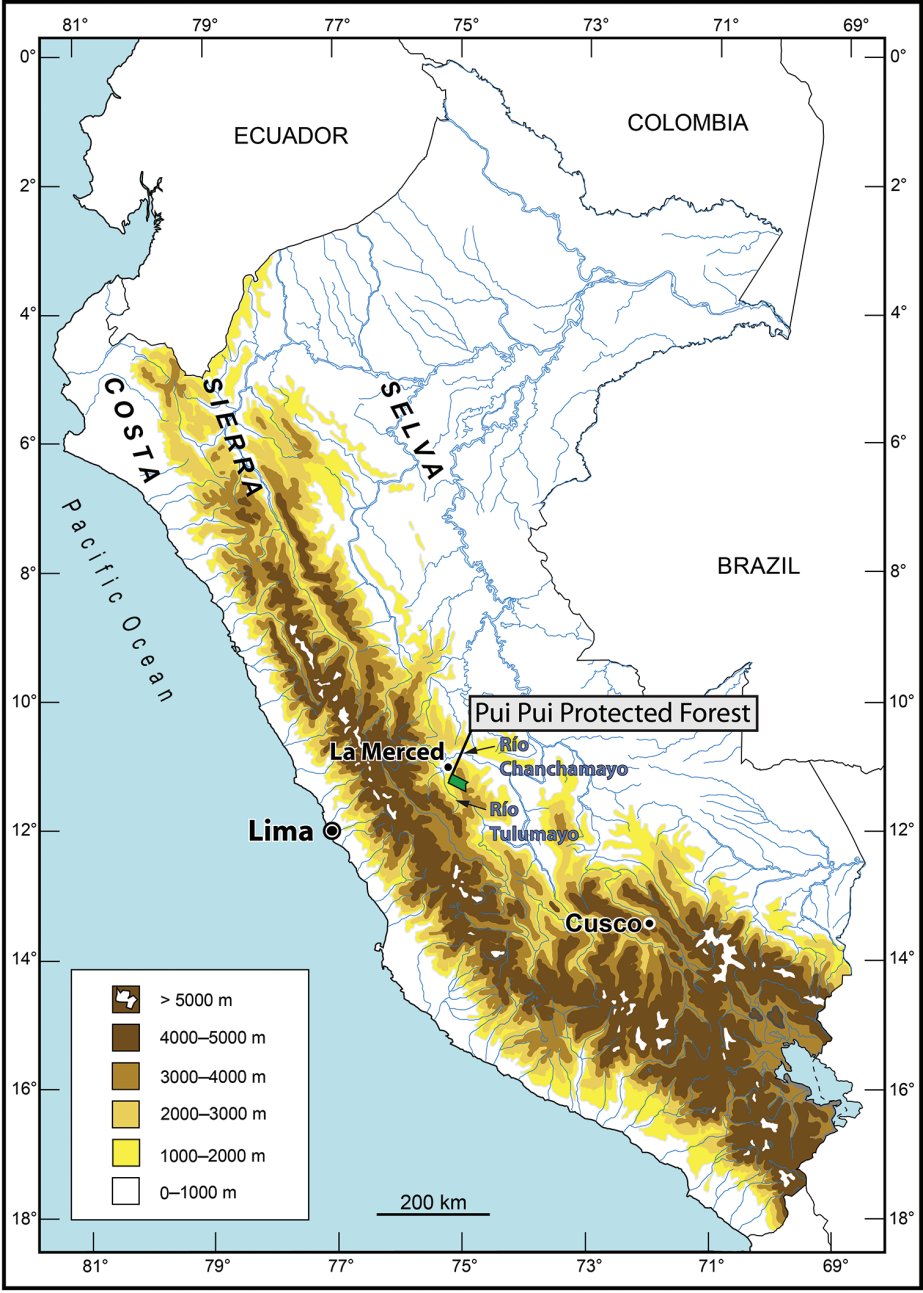
Map of Peru with the Pui Pui Protected Forest indicated in green.

The Pui Pui Protected Forest (Bosque de Protección Pui Pui, hereafter PPPF) is located in the Región Junín (Provincias Chanchamayo, Jauja, Concepción, and Satipo), was created 1985, and protects 60,000 hectares (30% montane forest, 70% puna habitats) between 1700 and 4500 m a.s.l. (Sernanp 2010). We surveyed the herpetofauna of the Pui Pui Protected Forest (Fig. 1) in montane forests and high Andean grasslands (puna) between 2012 and 2014 with the aim to record the amphibian and reptile species richness and to evaluate their conservation status. The collected specimens included several new species of craugastorid frogs. New species of craugastorid frogs are frequently discovered and scientifically described from Peru (e.g., Chávez and Catenazzi 2016, Padial et al. 2016, Shepack et al. 2016). Integrative taxonomy, the use of different character sets (e.g., morphological and molecular), has been very helpful to identify and describe new species (Padial et al. 2010) in this complicated group of frogs which has a high species diversity (487 species of which 127 occur in Peru, AmphibiaWeb 2016), cryptic diversity and often phenotypic polymorphism. Herein, we describe a new species of *Pristimantis* (*Pristimantis* sp. Pui Pui in Lehr et al. in press) from a montane forest between 1700 and 1800 m a.s.l in the PPPF.

## Methods

**Morphological characters.** The format for the description follows Lynch and Duellman (1997), except that the term dentigerous processes of vomers is used instead of vomerine odontophores (Duellman et al. 2006), and diagnostic characters are those of Duellman and Lehr (2009). Taxonomic classification follows Hedges et al. (2008) and Heinicke et al. (2009), except that we followed Pyron and Wiens (2011) for family placement, and Padial et al. (2014) for names of *Pristimantis* species groups. Specimens were fixed in 96% ethanol and stored in 70% ethanol. Liver tissues of three specimens (NMP6V 75063–65) were taken for genetic analyses (see Lehr et al. in press, GenBank accession numbers KY006110-12). Sex and maturity of specimens were identified through dissections of gonads. Specimens with a SVL ≤ 13.4 mm were considered juveniles when gonads were too small to distinguish between sexes. We measured the following variables to the nearest 0.1 mm with digital calipers under a stereomicroscope: snout–vent length (SVL, straight length distance from tip of snout to vent), tibia length (TL, distance from the knee to the distal end of the tibia), foot length (FL, distance from proximal margin of inner metatarsal tubercle to tip of Toe IV), head length (HL, from angle of jaw to tip of snout), head width (HW, at level of angle of jaw), horizontal eye diameter (ED), interorbital distance (IOD), upper eyelid width (EW), internarial distance (IND), eye–nostril distance (E–N, straight line distance between anterior corner of orbit and posterior margin of external nares). Fingers and toes are numbered preaxially to postaxially from I–IV and I–V, respectively. We compared the lengths of Toes III and V by adpressing both toes against Toe IV; lengths of Fingers I and II were compared by adpressing the fingers against each other. Drawings were made by EL using a stereomicroscope and a camera lucida. Photographs taken by EL and JM were used for descriptions of coloration in life. The 16S DNA barcoding was used for comparison of the new species with morphologically similar *Pristimantis* species from Ecuador and Peru (Lehr et al. in press). Comparisons of congeners focused on Andean and Amazonian lowland species from Ecuador and Peru with similar morphology and genetically close species as recovered in our tree (Lehr et al. in press). Information on species for comparative diagnoses was obtained from Duellman and Lehr (2009) and from original species descriptions. For specimens examined see Appendix 1. Codes of collections are: MUSM = Museo de Historia Natural Universidad Nacional Mayor de San Marcos, Lima, Peru; NMP6V = National Museum Prague, Prague, Czech Republic. Field number code is: IWU = Illinois Wesleyan University. Threat status was evaluated using the IUCN criteria (2016).

## Results

### Pristimantis ashaninka sp. n.

Taxon classificationAnimaliaAnuraCraugastoridae

http://zoobank.org/30AFB9BB-BCF4-4129-B63D-A7744E479E72

Suggested English name: Asháninka Rubber Frog

Suggested Spanish name: Rana cutín asháninka

**Figure 2.**
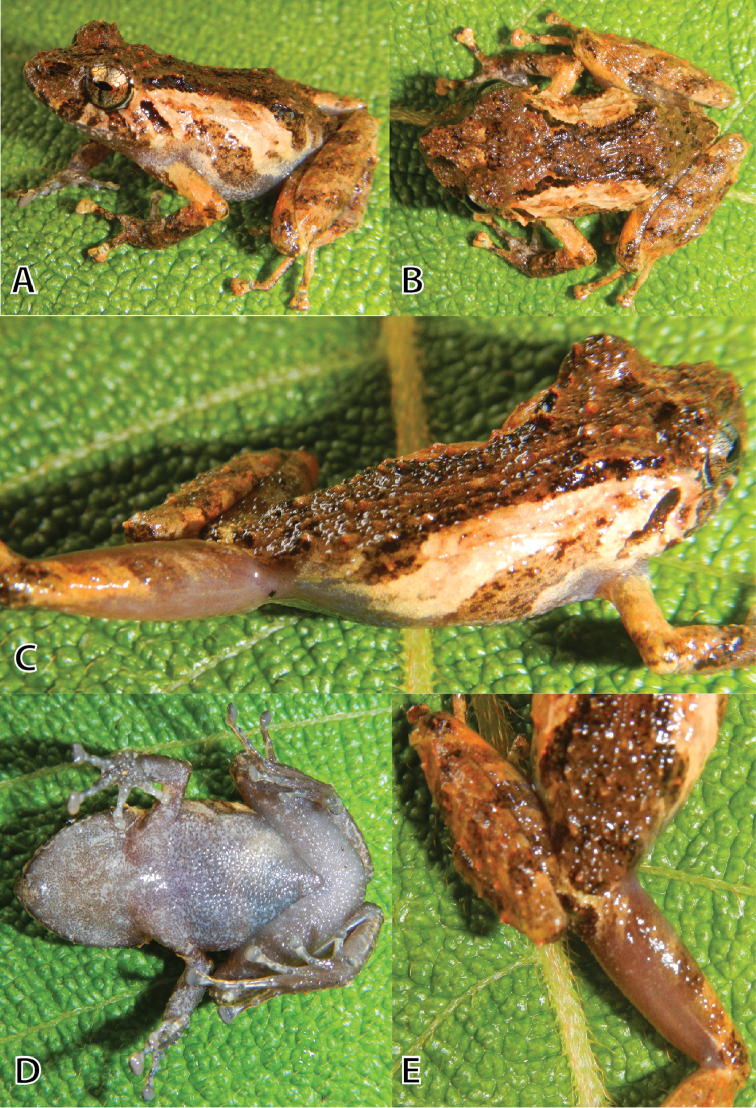
Life holotype (MUSM 36517, SVL 24.0 mm) of *Pristimantis ashaninka* sp. n. in lateral (**A**), dorsal (**B**), dorsolateral (**C**), ventral (**D**) views, and (**E**) posterior surface of thighs. Photos by E. Lehr.

Pristimantis sp. Pui Pui in Lehr et al. (in press, Fig. 2)

#### Holotype

**Figure 3.**
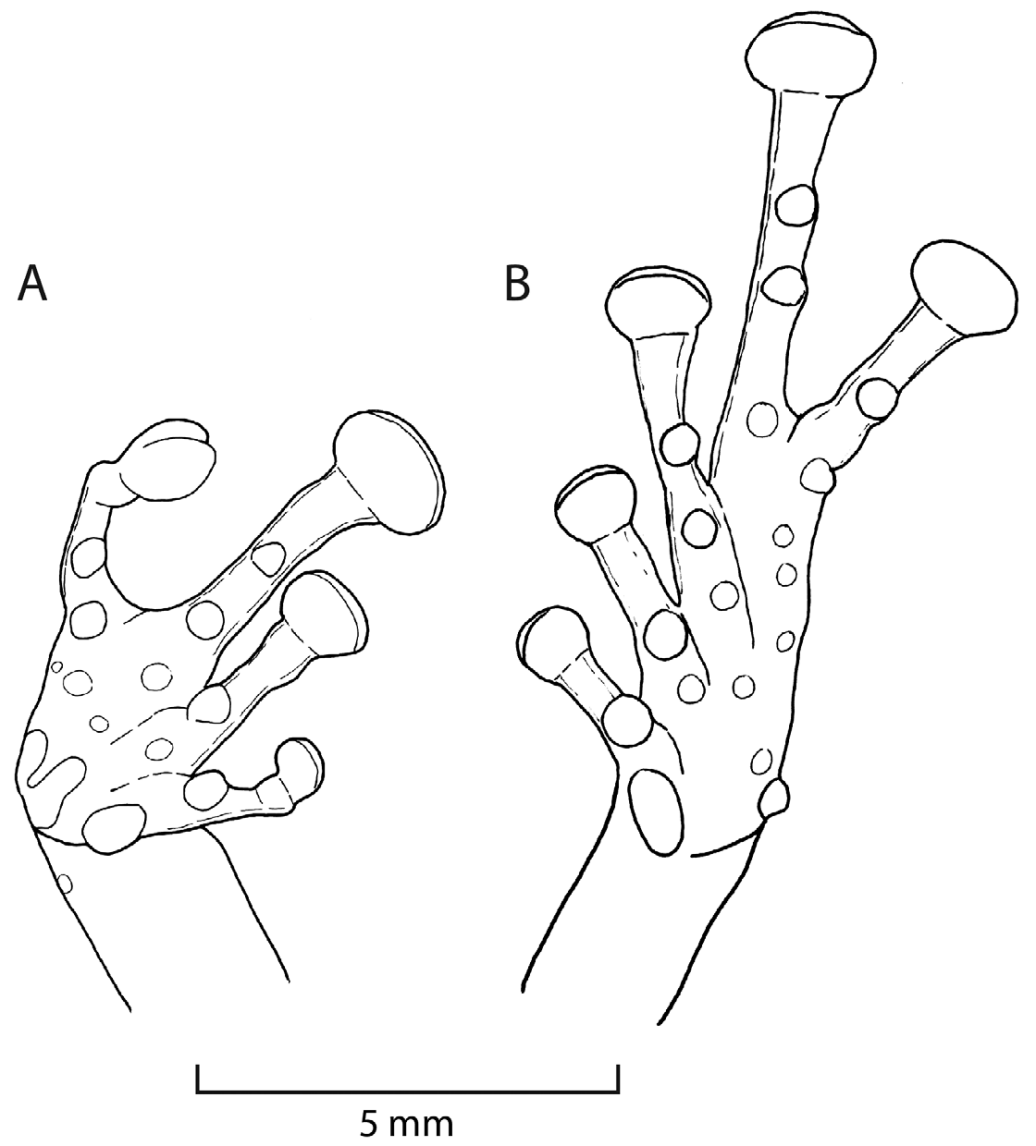
Ventral views of right hand (**A**) and left foot (**B**) of *Pristimantis ashaninka* sp. n. (holotype, MUSM 36517). Drawings by E. Lehr.

(Figs 2–3). MUSM 36517 (IWU 361), an adult female from the border of the Pui Pui Protected Forest (11°12'38.5"S, 74°57’28.9"W), 1700 m elevation, Distrito Pichanaqui, Provincia Chanchamayo, Región Junín, Peru, collected on 15 May 2014 by Edgar Lehr and Jiří Moravec.

#### Paratypes

**Figure 4.**
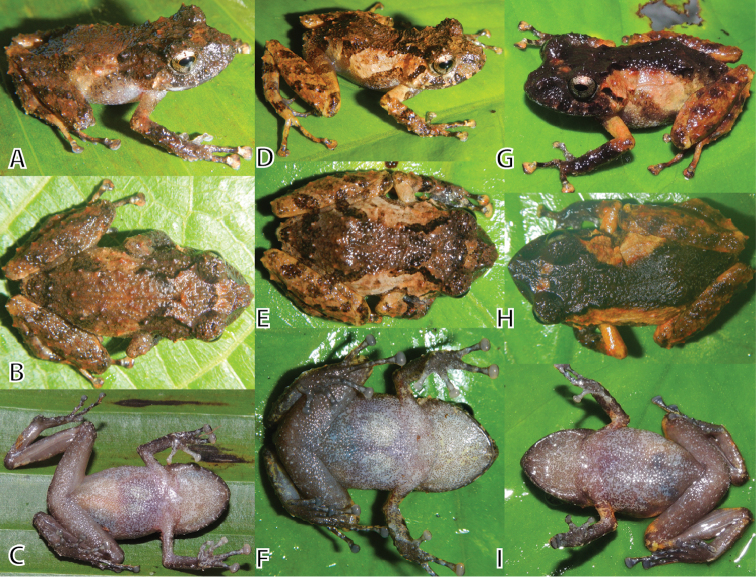
Female paratypes of *Pristimantis ashaninka* sp. n. in dorsolateral (upper row), dorsal (middle row), and ventral (lower row) views. **A–C** (MUSM 32736, SVL 23.1 mm) **D–F** (NMP6V 75063, SVL 26.7 mm) **G–I** (MUSM 32742, SVL 23.3 mm). Photos by E. Lehr (**A, B, D–F, H**) and J. Moravec (**C, G, I**).

**Figure 5.**
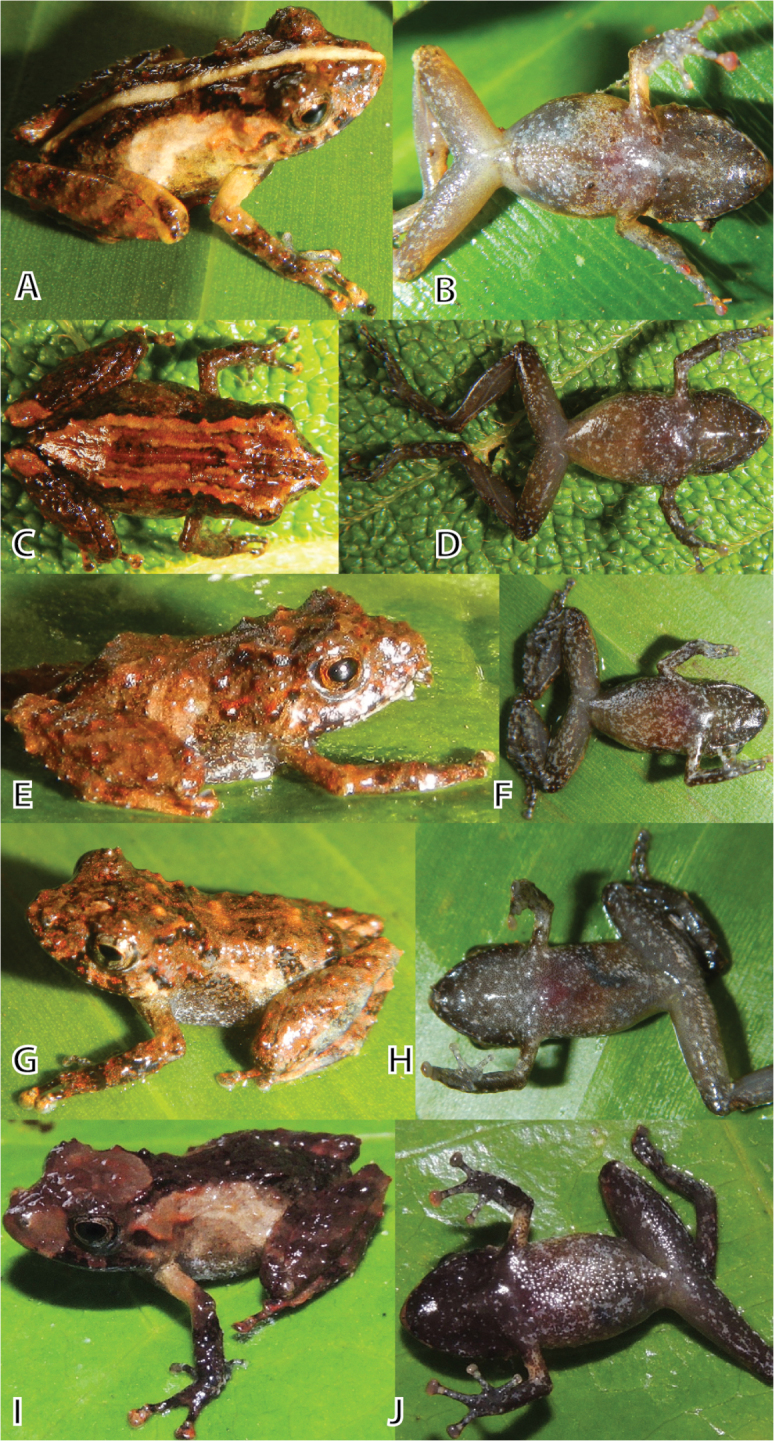
Juvenile paratypes of *Pristimantis ashaninka* sp. n. in dorsolateral/dorsal (left column) and ventral (right column) views. **A, B** (MUSM 32721, SVL 13.4 mm) **C, D** (MUSM 32730, SVL 12.6 mm) **E, F** (MUSM 32728, SVL 11.0 mm) **G, H** (MUSM 32722, SVL 12.5 mm), **I, J** (NMP6V 75555, SVL 12.1 mm). Photos by E. Lehr (**A–H**) and J. Moravec (**I, J**).

(Figs 4–5). Fourteen: four females: MUSM 32736, 32742, NMP6V 75063 (GenBank accession number KY006110), 75064 (KY006111); ten juveniles: NMP6V 75553–75555, MUSM 32721, 32722, 32728, 32730, 32734, 32741, NMP6V 75065 (KY006112), all collected at the type locality: on 13 May 2014 at 1700 m a.s.l. (NMP6V 75553, 75554, MUSM 32721, 32722, NMP6V 75065), on 14 May 2014 at 1700 m a.s.l. (MUSM 32728), on 15 May 2014 at 1700 m a.s.l. (MUSM 32730), on 17 May 2014 at 1700 m a.s.l. (MUSM 32734, NMP6V 75555), on 18 May 2014 at 1800 m a.s.l. (MUSM 32736, NMP6V 75064), on 20 May 2014 at 1750 m (MUSM 32741), on 21 May 2014 at 1800 m a.s.l. (MUSM 32742, NMP6V 75063).

#### Generic placement.

We assign this species to *Pristimantis* based on general morphological similarity to other members of the genus and our molecular data (Lehr et al. in press).

#### Diagnosis.

A new species of *Pristimantis* not assigned to any species group having the following combination of characters: (1) Skin on dorsum shagreen with many conical tubercles, skin on venter areolate; discoidal, thoracic, and dorsolateral folds absent; (2) tympanic membrane and tympanic annulus absent; (3) snout moderate, subacuminate in dorsal view, rounded in lateral view; (4) upper eyelid with enlarged conical tubercles; EW slightly shorter than IOD; cranial crests absent; (5) dentigerous processes of vomers present; (6) condition of vocal slits and nuptial pads unknown; (7) Finger I shorter than Finger II; discs of digits broadly expanded, rounded; (8) fingers with narrow lateral fringes; (9) small conical ulnar and tarsal tubercles present; (10) heel with small conical tubercles; inner tarsal fold absent; (11) inner metatarsal tubercle ovoid, 4 times as large as outer; outer metatarsal tubercle small, rounded; low, numerous supernumerary plantar tubercles; (12) toes with narrow lateral fringes; basal toe webbing absent; Toe V longer than Toe III; toe discs slightly smaller than those on fingers; (13) in life, dorsal coloration consists of a reddish-brown blotch in shape of a hourglass with dark grayish-brown markings, bordered laterally by creamish brown, a dark grayish-brown sinusoidal or W-mark on scapular region, extremities with dark grayish-brown bars; flanks usually paler than dorsum, creamish brown with broad diagonal dark grayish-brown stripes; dark grayish-brown bars on upper lip, dark grayish-brown canthal and supratympanic stripes present; groin, anterior and posterior surfaces of thighs uniformly grayish brown; venter pale gray and grayish brown mottled; iris pale bronze with fine black reticulations, a median reddish hint horizontally across iris, and a black narrow vertical streak from pupil across lower and upper half of iris; (14) SVL in adult females 23.1–26.7 mm (n = 5).

#### Comparisons.

*Pristimantis ashaninka* differs from its congeners by having the skin on dorsum shagreen with many conical tubercles giving it a spinose appearance, lacking a tympanum, having groin, anterior and posterior surfaces of thighs uniformly grayish brown, and a pale bronze iris with fine black reticulations, a median reddish hint horizontally across iris, and a black narrow vertical streak from pupil across lower and upper half of iris.

Seventeen species of *Pristimantis* from Peru lack a tympanum. These are *Pristimantis academicus* Lehr, Moravec & Gagliardi Urrutia, 2010, *Pristimantis altamazonicus* (Barbour & Dunn, 1921), *Pristimantis colodactylus* (Lynch, 1979), *Pristimantis coronatus* Lehr & Duellman, 2007, *Pristimantis croceoinguinis* (Lynch, 1968), *Pristimantis cruciocularis* (Lehr, Lundberg, Aguilar & von May, 2006), *Pristimantis flavobracatus* (Lehr, Lundberg, Aguilar & von May, 2006), *Pristimantis imitatrix* (Duellman, 1978), *Pristimantis lirellus* (Dwyer, 1995), *Pristimantis leucorrhinus* Boano, Mazzotti & Sindaco, 2008, *Pristimantis martiae* (Lynch, 1974), *Pristimantis minutulus* Duellman & Hedges, 2007, *Pristimantis rhabdocnemus* (Duellman & Hedges, 2005), *Pristimantis simonsii* (Boulenger, 1900), *Pristimantis tantanti* (Lehr, Torres-Gastello & Suárez-Segovia, 2007), *Pristimantis ventrimarmoratus* (Boulenger, 1912), and *Pristimantis vilcabambae* Lehr, 2007.

Of these species, *Pristimantis lirellus* from 470–1200 m a.s.l. (Dwyer 1995) on the eastern slopes of the Cordillera Central in northern Peru, *Pristimantis martiae* from Colombia, Ecuador to central Peru up to 1330 m a.s.l. (Lynch 1974), and *Pristimantis rhabdocnemus* from the Cordillera Oriental in central Peru between 230–2900 m a.s.l. (Duellman and Hedges 2005) are most similar regarding morphology and coloration to *Pristimantis ashaninka*. However, the 16S DNA barcoding revealed clear distinctions between all three species (sequences of *Pristimantis rhabdocnemus* include specimens from its type locality) and *Pristimantis ashaninka* (Lehr et al. in press). Furthermore, the new species can be distinguished from them as follows (characters of *Pristimantis ashaninka* in parentheses unless otherwise stated): *Pristimantis lirellus* is smaller (SVL in females 19.4–24.5 mm vs. 23.1–26.7 mm [n = 5] in *Pristimantis ashaninka*, Dwyer 2005), has skin on dorsum shagreen with small, scattered tubercles (many conical tubercles) and low longitudinal dermal ridge (absent), prominent discoidal fold (absent), groin with pale yellow to yellow-orange spot (uniformly grayish brown), and iris bronze to reddish brown with median horizontal red streak (pale bronze with fine black reticulations, a median reddish hint horizontally across iris, and a black narrow vertical streak from pupil across lower and upper half of iris). *Pristimantis martiae* and *Pristimantis ashaninka* have the dorsum brown with darker brown middorsal blotch in shape of a hourglass and pale brown flanks, but *Pristimantis martiae* is smaller (SVL in females 18.3–23.00 mm, Lynch 1974), has skin on dorsum shagreen with low, flat warts (shagreen with many conical tubercles), groin, anterior and posterior surfaces of thighs dull cream or pale orange with brown to black bars or mottling (uniformly grayish brown), and a bronze iris with median horizontal brown streak (pale bronze with fine black reticulations, a median reddish hint horizontally across iris, and a black narrow vertical streak from pupil across lower and upper half of iris). *Pristimantis rhabdocnemus* is of similar size (SVL in females 23.2–27.0 mm, Duellman and Hedges 2005), has skin on dorsum shagreen with or without scattered small tubercles (shagreen with many conical tubercles), posterior surfaces of thighs tan (uniformly grayish brown), and iris grayish tan (pale bronze with fine black reticulations, a median reddish hint horizontally across iris, and a black narrow vertical streak from pupil across lower and upper half of iris).

*Pristimantis platydactylus* (Boulenger, 1903) and *Pristimantis wiensi* (Duellman and Wild, 1993) look superficially similar with *Pristimantis ashaninka*, but can be distinguished as follows. *Pristimantis platydactylus* from the eastern Andes of central Peru to central Bolivia at elevations between 930 and 3470 m a.s.l. (Duellman and Lehr 2009) contains several unnamed species (Padial et al. 2009), is much larger (SVL in females 23.8–35.3 mm, Duellman and Lehr 2009), has a tympanum (absent), and is genetically different (Lehr et al. in press) based on sequences from 14 specimens of *Pristimantis platydactylus* from Peru and Bolivia obtained from Padial et al. (2009). *Pristimantis wiensi* from northern Peru (western slopes of Cordillera Huancabamba between 1600 and 1735 m a.s.l., Duellman and Wild 1993) is much larger (SVL in single female 37.0 mm, Duellman and Wild 1993), has discoidal and dorsolateral folds (both absent), and has a tympanum (absent).

Furthermore, *Pristimantis ashaninka* differs from other Andean *Pristimantis* (in alphabetical order) that lack a tympanum as follows: *Pristimantis colodactylus* from southern Ecuador and northern Peru between 2195–3140 m a.s.l. (Lynch 1979) has short and stocky fingers (of normal length) with small discs (broad). *Pristimantis coronotus* from northern Peru at 2850 m a.s.l. (Lehr and Duellman 2007) has posterior half of flanks, groin and proximal anterior surfaces of thighs red (uniformly grayish brown). *Pristimantis cruciocularis* from 1330–1850 m a.s.l. in the Cordillera Oriental in central Peru is smaller (SVL in females 18.7–21.8 mm, Lehr et al. 2006), has groin and anterior surfaces of thighs orange to red (uniformly grayish brown), and iris golden with fine black reticulations and dark brown horizontal and vertical streaks forming a cross (pale bronze with fine black reticulations, a median reddish hint horizontally across iris, and a black narrow vertical streak from pupil across lower and upper half of iris). *Pristimantis flavobracatus* from the Amazonian slopes of the Cordillera Oriental at 1770 m a.s.l. in central Peru is smaller (SVL in females 21.5–23.4 mm, Lehr et al. 2006), has groin and anterior and posterior surfaces of thighs yellow (uniformly grayish brown), and golden iris with fine black reticulations with a brown median horizontal streak across iris and a black vertical streak downward from pupil (pale bronze with fine black reticulations, a median reddish hint horizontally across iris, and a black narrow vertical streak from pupil across lower and upper half of iris). *Pristimantis leucorrhinus* from the Cordillera Oriental in central Peru at 2500 m a.s.l. (Boano et al. 2008) has the upper eyelid with one large conical tubercle (enlarged conical tubercles, but not as large as in *Pristimantis leucorrhinus*), a black groin (uniformly grayish brown) and anterior surfaces of thighs black (uniformly grayish brown) with two large white spots on each. *Pristimantis minutulus* from the Cordillera Oriental and lowland forests in central Peru between 250 and 1200 m a.s.l. (Duellman and Hedges 2007) has the groin with a large yellow blotch (uniformly grayish brown). *Pristimantis simonsii* from the Cordillera Occidental in northern Peru at elevations of 3050–3760 m a.s.l. (Duellman and Lehr 2009) has dorsolateral folds (absent). *Pristimantis vilcabambae* from the Cordillera de Vilcabamba at 2050 m a.s.l. (Lehr 2007) in southern Peru has fingers and toes with distinct lateral fringes (narrow), outer fringes of Finger IV and Toe V often continuing as discontinuous fold to outer edge of palm or plantar (absent), and venter cream with dark brown blotches (venter pale gray and grayish brown mottled).

Furthermore, *Pristimantis ashaninka* differs from the Amazonian lowland *Pristimantis* (in alphabetical order) that lack a tympanum as follows: *Pristimantis academicus* from lowlands of northern Peru is smaller (20.0–22.0 mm, Lehr et al. 2010), and has a yellow groin (uniformly grayish brown). *Pristimantis altamazonicus* from southern Colombia, Ecuador, Peru, and western Brazil has the groin, anterior and posterior surfaces of thighs red to salmon color with black mottling (uniformly grayish brown). *Pristimantis croceoinguinis* from southern Colombia, Ecuador, and extreme northeastern Peru is smaller (SVL in females 17.4–23.0 mm, Lynch 1968), has skin on dorsum tuberculate (shagreen with many conical tubercles), supernumerary plantar tubercles absent (present), canthal and postorbital stripes absent (present), deep yellow to orange spot in groin (groin uniformly grayish brown), and iris dull bronze with dense brown reticulations (pale bronze with fine black reticulations, a median reddish hint horizontally across iris, and a black narrow vertical streak from pupil across lower and upper half of iris). *Pristimantis imitatrix* from central and southern Peru and *Pristimantis ashaninka* have skin on dorsum shagreen with conical tubercles and bronze iris with median horizontal red streak, but *Pristimantis imitatrix* is much smaller (SVL in females 14.6–20.2 mm, Duellman 1978), has dentigerous processes of vomers absent (present), supernumerary plantar tubercles absent (present), and groin, anterior and posterior surfaces of thighs mottled black and creamy white (uniformly grayish brown). *Pristimantis tantanti* from southern Peru has short triangular snout (subacuminate), and green dorsum with white spots (reddish brown with dark grayish-brown markings). *Pristimantis ventrimarmoratus* from the upper Amazon Basin and slopes of the Andes to 1740 m in Ecuador, Peru, and Bolivia (Duellman and Lehr 2009) has prominent discoidal fold (absent), and chest and belly white with bold black mottling (venter pale gray and grayish brown mottled).

#### Description of holotype.

Head slightly narrower than body, slightly longer as wide; head length 39% of SVL; head width 38% of SVL; cranial crests absent; snout moderately long, subacuminate in dorsal view, rounded in lateral view (Fig. 2A, B); eye-nostril distance 77% of eye diameter; nostrils slightly protuberant, directed dorsolaterally; canthus rostralis moderately long, rounded in lateral view, weakly concave in dorsal view; loreal region concave; lips rounded; upper eyelid each with several tubercles, an enlarged conical tubercle at its center and one enlarged conical tubercle at its posterior end; upper eyelid width 90% of IOD; several enlarged conical tubercles on occipital and scapular region (see photos in life Fig. 2B, C); supratympanic fold short and broad, extending from posterior margin of upper eyelid slightly curved to insertion of arm; tympanic membrane and annulus absent; two conical postrictal tubercles present bilaterally. Choanae small, ovoid, not concealed by palatal shelf of maxilla; dentigerous processes of vomers small, widely separated; tongue discoidal, covering entire floor of mouth, posterior and lateral parts free.

Skin on dorsum and flanks shagreen with many conical tubercles (denser on dorsum than on flanks), dorsolateral folds absent; skin on throat and chest smooth, on belly areolate; discoidal and thoracic folds absent; cloacal sheath short.

Outer ulnar surface with minute low tubercles; palmar tubercle partially divided distally; thenar tubercle ovoid; subarticular tubercles well defined, round in ventral view, conical in lateral view; supernumerary tubercles distinct, ovoid, subconical, approximately half the size of subarticular tubercles; fingers with narrow, weakly defined lateral fringes; Finger I shorter than Finger II; discs on digits of fingers widely expanded, truncate (Fig. 3A).

Hind limbs moderately long, slender, tibia length 54% of SVL; foot length 46% of SVL; upper surfaces of hind limbs smooth with scattered tubercles; inner surface of thighs smooth, posterior and ventral surfaces of thighs weakly areolate; heels each with two prominent conical tubercles; outer surface of tarsus with scattered minute low tubercles; inner tarsal fold absent; inner metatarsal tubercle prominent, ovoid, four times the size of round outer metatarsal tubercle; subarticular tubercles well defined, round in ventral view, conical in lateral view; plantar supernumerary tubercles distinct, about quarter the size of subarticular tubercles; toes with narrow, weakly defined lateral fringes; basal webbing absent; discs expanded, oval, less expanded than those on fingers; relative length of toes: 1<2<3<5<4; disc on Toe III not reaching distal subarticular tubercle on Toe IV, disc on Toe V extends distal subarticular tubercle on Toe IV; Fig. 3B.

#### Measurements of the holotype

**(in mm).** SVL 24.0; TL 13.0; FL 11.0; HL 9.4; HW 9.2; ED 3.1; IOD 3.0; EW 2.7; IND 1.9; E-N 2.4.

#### Coloration of the holotype in life

**(Fig. 2).** Dorsum covered by a large reddish-brown blotch in shape of a hourglass bordered laterally in its narrow portion by creamish brown coloration; a dark grayish-brown W-mark on scapular region covering the anterior portion of the hourglass; two longitudinal oblique dark grayish-brown markings on sacral region bordering the posterior portion of the hourglass; a small dark grayish-brown marking on intercanthal region; a diffuse dark grayish-brown to reddish-brown interorbital stripe; a diffuse dark grayish-brown blotch on canthus rostralis; upper lip with two reddish-brown subocular bars bordered by dark grayish-brown on each side of the head; dark grayish-brown spots in front of and in between subocular bars; a dark grayish-brown supratympanic bar; upper arm creamish brown dorsally; lower arm and hand reddish-brown dorsally except for creamish-brown discs; hind legs creamish brown dorsally with pale reddish-brown diagonal bars; toes creamish brown; flanks creamish brown with pale reddish-brown diagonal blotches; axilla, groin, and anterior and posterior surfaces of thighs uniformly grayish brown; throat, chest, belly, and ventral surfaces of thighs, hands, and feet pale gray and grayish brown mottled; iris pale bronze with fine black reticulations, a median reddish hint horizontally across iris, and a black narrow vertical streak from pupil across lower half of iris and thin dark gray streak across upper half of iris.

#### Coloration of the holotype in preservative.

General coloration pattern is as described for the holotype in life, except for reddish brown which is dark brown and creamish brown which is pale tan. Groin and axilla are pale gray, anterior and posterior surfaces of thighs are pale brown; ventral surfaces except for brown thighs are pale brown and pale tan mottled; iris is silver with fine black reticulations with black narrow vertical streak from pupil across lower half of iris and thin dark gray streak across upper half of iris.

#### Variation.

All female paratypes (Fig. 4, SVL 23.1–26.7, 24.1 ± 1.5, n = 4) and juveniles (Fig. 5, SVL 10.6–13.4, 12.1 ± 1.0, n = 10) are similar to the holotype regarding morphology (Tables 1, 2) and coloration pattern. Two females (MUSM 32742, NMP6V 75063, Fig. 4E, H) have dorsally an hourglass shape blotch as seen in the holotype with the hourglass dark brown in MUSM 32742, two other females (MUSM 32736, NMP6V 75064) lack the coloration contrast of dark dorsum and pale flanks. One female (NMP6V 75064) has the anterior interorbital and snout region creamish brown, three females (MUSM 32736, 32742, NMP6V 75063) have the interorbital area with two creamish brown or reddish brown blotches (Fig. 4B, E, H). Finger and toe discs are dorsally creamish brown (Fig. 4A, D, G).

**Table 1.** Measurements (in mm) of female type specimens of *Pristimantis ashaninka* sp. n. For abbreviations see methods.

Character	NMP6V 75063	MUSM 36517	MUSM 32742	NMP6V 75064	MUSM 32736
SVL	26.7	24.0	23.3	23.2	23.1
TL	13.6	13.0	12.5	12.8	13.0
FL	11.6	11.0	9.9	10.6	10.9
HL	10.7	9.4	9.4	9.4	9.6
HW	10.4	9.2	9.3	9.0	8.9
ED	3.6	3.1	2.8	3.3	2.7
IOD	3.2	3.0	2.5	2.7	2.8
EW	2.8	2.7	2.2	2.6	2.7
IND	2.5	1.9	1.8	2.1	2.0
E–N	2.7	2.4	2.3	2.4	2.3

**Table 2.** Measurements (in mm) and proportions of female type specimens of *Pristimantis ashaninka* sp. n.; ranges followed by means and one standard deviation in parentheses. For abbreviations see methods.

Characters	Females (n = 5)
SVL	23.1–26.7 (24.1 ± 1.4)
TL	12.5–13.6 (13.0 ± 0.4)
FL	9.9–11.6 (10.8 ± 0.6)
HL	9.4–10.7 (9.7 ± 0.5)
HW	8.9–10.4 (9.4 ± 0.5)
ED	2.7–3.6 (3.1 ± 0.3)
IOD	2.5–3.2 (2.8 ± 0.2)
EW	2.6–2.8 (2.6 ± 0.2)
IND	1.8–2.5 (2.1 ± 0.2)
E–N	2.3–2.7 (2.4 ± 0.1)
TL/SVL	0.51–0.56
FL/SVL	0.42–0.47
HL/SVL	0.39–0.42
HW/SVL	0.38–0.40
HW/HL	0.9–1.0
E–N/ED	0.73–0.85
EW/IOD	0.88–0.96

The juveniles have the conical tubercles on the dorsal skin more pronounced (MUSM 32722, 32741, Fig. 5G) than the females. Conical tubercles form ridges on scapular region (MUSM 32722, 32728, 32741, Fig. 5E, G) and laterally on head and flanks (NMP6V 75555, Fig. 5I). The coloration pattern is similar, but juveniles seem to have a more reddish hint especially dorsally and laterally on head and scapular region (MUSM 32722, 32728, 32730, 32741, NMP6V 75554, Fig. 5E, G). One specimen (MUSM 32721, Fig. 5A) has a pale tan middorsal stripe dividing the dark reddish brown hourglass blotch. One specimen (MUSM 32730, Fig. 5C) has heels and dorsum pale reddish brown with dark reddish-brown flecks on dorsum. One specimen (NMP6V 75555, Fig. 5I) has the head dorsally and snout dorsally and laterally pale grayish brown. Ventral coloration of juveniles (Fig. 5, right column) is darker than in females with pale gray flecks, with the throat often black (NMP6V 75555) to dark grayish brown (MUSM 32728, 32730) with pale gray flecks. Finger and toe discs are dorsally creamish brown.

#### Etymology.

The species epithet *ashaninka* is used in reference to the indigenous people Asháninka who inhabit forests in the Peruvian Regions Huánuco, Junín, Pasco, and Ucayali.

#### Distribution, natural history, and threat status.

*Pristimantis ashaninka* is only known from the type locality, which is located at the northeastern border of the Pui Pui Protected Forest, ca. 18 km (straight airline) NW of the town of Satipo, Distrito de Pichanaqui, Privincia de Chanchamayo, Región Junín, Peru (Fig. 1). The type locality lies in the valley of a tributary of the Rio Bravo at an elevation between 1700 and 1800 m a.s.l. and can be reached by walking in one and a half day starting at the village of Ayte (11°09‘46.7‘‘S, 74°55‘14‘‘W, 1295 m a.s.l.), which serves as a control station for the administration of the PPPF. The valley is surrounded by steep mountain slopes, which gives it a narrow character. The surrounding mountains are covered by a primary mountain rainforest characterized by 15–20 m high canopy and frequent occurrence of bromeliads, ferns, and epiphytic mosses. The adult specimens of *Pristimantis ashaninka* were collected at night on vegetation up to 150 cm above the ground whereas juveniles occupied lower positions in the vegetation. Other craugastorid species found at the type locality in sympatry with *Pristimantis ashaninka* included *Pristimantis* cf. *albertus*, *Pristimantis bipunctatus* (Duellman and Hedges, 2005), *Pristimantis cruciocularis*, *Pristimantis* cf. *platydactylus*, and *Pristimantis* sp. nov. According to the sparse data available, we here classify *Pristimantis ashaninka* as “Data Deficient” according to the IUCN red list criteria.

## Discussion

Despite high species diversity and endemism, many areas throughout the Tropical Andes remain unexplored because montane forests are difficult to reach. Consequently, biodiversity in these areas is poorly known compared to other ecoregions at lower elevations. This was true for the Pui Pui Protected Forest, which is located in the eastern Andes of the Region Junín. Because of its remote location and difficult access no biological surveys have been conducted inside the PPPF prior to our expeditions. The borders of the PPPF are covered by primary mountain forests interrupted with scattered coffee plantations and a few houses along the larger rivers. New road constructions to support expanding villages in the region are a latent threat to these cloud forests, which are considered valuable for some of their timber trees or as new land for agricultural crops such as passionfruit (*granadilla*) and chili pepper (*rocoto*). Contemporary plans to construct a dam with a power station on Rio Huatziroki in the northern border of the PPPF are an additional threat. This example points to the great importance of the existing buffer zone of PPPF, which primary role to preventing habitat destructions around the PPPF borders should be maximally respected. Given that the only known locality of *Pristimantis ashaninka* lies outside the boundary of the PPPF, the long-term protection of this species will depend on the type of land use in the area. This is especially relevant considering that large areas of potentially suitable habitat have been converted to agriculture outside the preserve. Given that many amphibian species, including dozens of threatened species, in Peru are known to occur only outside natural protected areas (von May et al. 2008), is it essential to carry out additional field surveys focusing on target species to determine if their populations occur inside protected areas.

With the description of *Pristimantis ashaninka*, the number of *Pristimantis* known from Peru rises to 128 species (AmphibiaWeb 2016). Further new species of *Pristimantis* and *Phrynopus* from the PPPF and its surroundings will be described in the near future.

## Supplementary Material

XML Treatment for Pristimantis ashaninka

## Appendix 1

**Comparative specimens examined**

*Pristimantis* cf. *albertus* (1 specimen): PERU: Junín: PPPF, 11°12'38.5"S, 74°57'28.9"W, 1700 m, MUSM 32729.

*Pristimantis bipunctatus* (2 specimens): PERU: Junín: PPPF, 11°12'38.5"S, 74°57'28.9"W, 1700 m, MUSM 32723, 32724.

*Pristimantis cruciocularis* (2 specimens): PERU: Pasco: Yanachaga-Chemillén National Park (Sector San Daniel), Distrito de Huancabamba, Provincia de Oxapampa, ca. 2900 m, MUSM 31140, 31146.

*Pristimantis imitatrix* (2 specimens): PERU: Madre de Dios: Cusco Amazónico, 15 km E Puerto Maldonado, 200 m, MUSM 7348, 14605.

*Pristimantis lirellus* (1 specimen): PERU: Huánuco: Dantas, MUSM 11326.

*Pristimantis minutulus* (10 specimens): PERU: Huánuco: Panguana, SMNS 13017–22; Pasco: 0.0–1.5 km W Cacazu, 900 m, KU 291677, 291679, 291680, 308608.

*Pristimantis* cf. *platydactylus* (3 specimens): PERU: Junín: PPPF, 11°12'38.5"S, 74°57'28.9"W, 1800 m, MUSM 32735; Peru, 11°05'44.2"S, 75°13'39.8"W, 1550 m, MUSM 31929, 31930.

*Pristimantis rhabdocnemus* (3 specimens): PERU: Pasco: ca. 10°23’.718 S, 75°28’.919 W, 2350 m, MUSM 31110, 31112, 31114.
